# The triple-hit hypothesis of Alzheimer’s disease: blood–brain barrier breakdown, infection, and neuroimmune activation as a unified etiological framework

**DOI:** 10.3389/fnins.2026.1815976

**Published:** 2026-05-22

**Authors:** Gregor Majdic

**Affiliations:** Veterinary Faculty, University of Ljubljana, Ljubljana, Slovenia

**Keywords:** Alzheimer’s disease, amyloid beta, blood–brain barrier, brain, neuroinflammation

## Abstract

Studies of Alzheimer’s disease (AD) have long been dominated by the amyloid cascade hypothesis, although mounting evidence suggests that amyloid-β (Aβ) deposition might be a late downstream event, rather than the initiating trigger of AD. Here, I propose a unifying *Triple-Hit Hypothesis* in which AD develops through a sequential interaction among three causative processes that have been individually implicated before in the onset of Alzheimer’s disease: (1) early blood–brain barrier (BBB) breakdown, (2) entry or reactivation of microbial agents within the brain, and (3) maladaptive innate immune responses that produce chronic neuroinflammation and Aβ accumulation. Converging data from human imaging, neuropathology, infectious disease studies, genetics, and vascular medicine could suggest that these three processes, which were in some previous studies linked to AD, might not be independent but rather that their temporal synergy could drive disease progression. This new conceptual framework integrates long-standing but fragmented lines of evidence and, if confirmed by experimental studies, offers the possibility for the development of new diagnostic biomarkers, new therapeutic entry points, and prevention strategies for AD. I argue that understanding AD as a disorder of the blood-brain barrier, immunity, and host–pathogen interactions should be taken into account in future research on the etiology and clinical progression of AD.

## 1 Introduction: Why we need a new framework for Alzheimer’s disease?

For over three decades, Alzheimer’s disease (AD) studies have been dominated by the amyloid cascade hypothesis, which suggests that misfolded amyloid-β (Aβ) plaques initiate a downstream sequence of neurodegenerative events, likely together with neurofibrillary tangles composed of hyperphosphorylated tau proteins. Yet several observations challenge this view. Numerous studies have shown that: (a) Aβ burden correlates poorly with cognitive decline; (b) older individuals with abundant plaques are often not cognitively impaired; (c) some anti-amyloid therapies can remove plaques but show limited clinical benefit; and (d) early vascular changes, inflammation, and metabolic dysfunction appear decades before amyloid accumulation detectable on PET imaging (Knopman et al., 2021), although neuronal damage by Aβ plaques is almost certainly responsible for early cognitive decline, but the accumulation of Aβ is probably too low to be detected by current diagnostic tools.

Simultaneously, findings that were historically considered peripheral—changes in blood–brain barrier (BBB) permeability, presence of various infectious agents in the brain, and microglial overactivation—have accumulated over the years, although they remain poorly integrated into a coherent etiological framework.

Here, I propose a conceptual, novel model of AD etiology, the Triple-Hit Hypothesis, which proposes a unifying sequence of harmful events leading to the onset of AD. Although all three events have been individually proposed to contribute to the onset of AD, this is the first conceptual model combining all three harmful events into a single etiology model. The first event in this novel hypothesis is the breakdown or increased permeability of the BBB. This would allow entry of pathogens (and perhaps endogenous immune molecules) into the brain, followed by an exaggerated immune response that triggers Aβ accumulation and the formation of neurofibrillary tangles, leading to neurodegeneration and ultimately to the development of AD symptoms.

**FIGURE 1:**
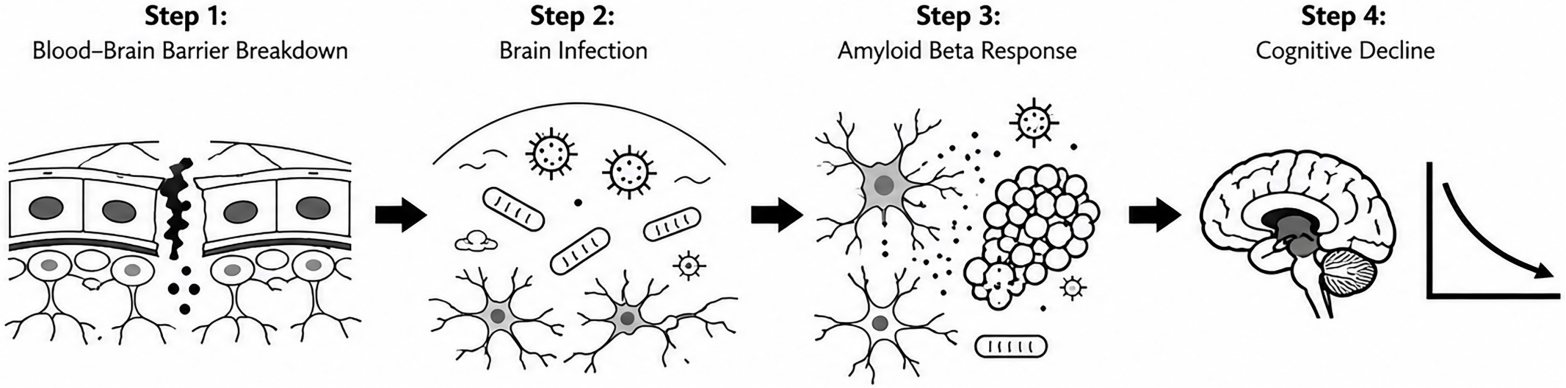
Schematic representation of proposed temporal events leading to the development of Alzheimer’s disease.

The Triple-Hit Hypothesis builds upon previous studies/models of Alzheimer’s disease that suggested microbial factors, neuroinflammation, and blood–brain barrier (BBB) dysfunction (Fulop et al., 2018). Rather than proposing entirely new components, the key innovation of this hypothesis lies in the explicit temporal ordering of these events. Specifically, I propose that early BBB disruption represents a primary event that facilitates subsequent microbial entry and amplifies neuroinflammatory responses. I recognize that alternative scenarios have been suggested, including models in which infection or systemic inflammation precede BBB impairment, and experimental data (e.g., LPS-induced models; Zhao et al., 2019) support such possibilities. However, given emerging evidence that BBB dysfunction may occur early in the disease process and the fact that many diseases that cause disruption of the BBB are very prominent risk factors for AD, I propose that this sequence represents a plausible and experimentally testable framework. Importantly, this temporal perspective allows for clearer hypothesis-driven investigation of causality in Alzheimer’s disease pathogenesis (Figure 1).

This novel perspective incorporates vascular biology, infectious disease, and neuroimmunology into a single model, suitable to explain a broad range of clinical, imaging, and genetic results regarding AD.

## 2 The first hit: blood–brain barrier breakdown as the earliest detectable event

Blood–brain barrier integrity declines with age, but some studies suggest that in AD-prone individuals, this deterioration might occur unusually early. Several human imaging studies using dynamic contrast-enhanced MRI have shown BBB leakage in the hippocampus and parahippocampal regions in cognitively normal adults who later develop cognitive impairment (Nation et al., 2019). Similarly, carriers of certain APOE4 alleles, which confer increased susceptibility to AD, demonstrate BBB breakdown in the hippocampus and medial temporal lobe independently of amyloid or tau pathology, beginning as early as the fourth decade of life (Montagne et al., 2020).

Additional evidence for contributions of BBB breakdown or increased permeability in AD etiology includes elevated CSF/serum albumin ratio in preclinical AD (Skoog et al., 1998), early reductions in tight junction proteins such as claudin-5 and occludin (Yamazaki and Kanekiyo, 2017), as well as pericyte degeneration and loss of vascular mural cells in patients with AD (Sengillo et al., 2013). Together, these findings suggest that BBB breakdown might not be secondary to amyloid or tau pathology but could potentially precede them, and may be involved in early processes leading to the onset of clinical AD, although further studies will be needed to firmly confirm this.

In addition to age, several other biological and pathological processes are known to weaken the BBB. These include metabolic disorders such as diabetes and insulin resistance, which disrupt endothelial function and increase inflammatory signaling (Biessels et al., 2008); hypertension, which induces mechanical stress and microvascular rarefaction (Faraco and Iadecola, 2013); chronic kidney disease, which elevates circulating toxins that impair endothelial tight junctions (Sarnak et al., 2003); and aging itself, which reduces pericyte coverage and astrocytic endfoot function, leading to impaired regulation of cerebral blood flow and barrier permeability (Verheggen et al., 2020). Interestingly, the study by Biessels et al. (2008) suggests that the connection between diabetes and cognitive decline is mostly associated with old age or when diabetes presents with micro- or macrovascular lesions, further suggesting the vascular component to the development of cognitive decline. These pathological processes all align with epidemiological studies demonstrating that vascular and metabolic disorders account for up to 40% of AD risk, while aging is a major factor contributing to disease onset.

The BBB serves as the brain’s primary defense against pathogens and systemic inflammatory signals. Its failure enables entry of viruses or dormant pathogens, migration of bacteria or bacterial fragments, increased penetration of peripheral cytokines, and infiltration of immune cells such as neutrophils and monocytes into the brain. BBB breakdown or increased permeability will thus be essential in enabling the “second hit” in the Triple-Hit Hypothesis.

## 3 The second hit: microbial invasion or viral reactivation

In the intact state, the BBB prevents most pathogens from entering the CNS. However, when the barrier is compromised, circulating viruses can cross into the brain parenchyma, especially during systemic reactivation, and latent neurotropic viruses may reactivate in response to inflammation or stress. Furthermore, bacterial fragments like LPS can diffuse into the CNS through compromised BBB, even without live bacterial invasion, and such microbial metabolites can trigger localized innate immune activation (Galea, 2021).

In recent years, several studies have reported microbial signatures in post-mortem brain tissue from AD patients, although these are not yet generally accepted, and not all studies have been sufficiently replicated, so the role of infection in the etiology of AD remains speculative (Zhao et al., 2024). These include herpes simplex virus type 1 (HSV-1), human herpesviruses 6 and 7, and bacteria such as *Chlamydia pneumoniae* and *Porphyromonas gingivalis* (Dominy et al., 2019; Itzhaki et al., 1997; Readhead et al., 2018). HSV-1 has been detected in the brains of many older adults and is significantly associated with AD, especially in APOE4 carriers, highlighting a potential connection between BBB dysfunction and infection in the etiology of AD (Itzhaki and Lathe, 2018; Linard et al., 2020). Herpesviruses have been detected in the AD hippocampus and temporal cortex (Yong et al., 2021), and HSV-1 and varicella-zoster virus infection have been associated with increased risk for AD (Shin et al., 2024). Although not all results are universally replicated, collectively these results do suggest that microbial agents might be a common feature in AD brains and may contribute to disease development if they gain access into the brain due to BBB disruption. However, the role of infection in AD is not yet generally accepted, and further clinical and postmortem studies will be needed to firmly establish potential connections between infection and the etiology of AD. Furthermore, there is also a possibility of certain microorganisms entering the brain directly through neural pathways like trigeminal nerve or even craniofacial nerves, thus bypassing BBB. This could suggest that even if triple-hit hypothesis will be confirmed in the future studies as the main etiology of AD, AD will likely remain a heterogenous disease with additional possible harmful events at all three crucial steps.

Several animal and *in vitro* studies have also demonstrated that HSV-1 infection increases Aβ production and induces tau phosphorylation (Eimer et al., 2018). Bacterial LPS inoculation induces microglial activation and tau hyperphosphorylation (Bhaskar et al., 2010), and repeated systemic infections worsen cognition in amyloid-prone mice (Krstic et al., 2012). In humans, some epidemiological studies have shown that antiviral treatments reduce the risk of dementia in individuals with recurrent HSV infections (Tzeng et al., 2018).

According to the triple hit hypothesis, the BBB normally protects the brain from infection, and therefore, peripheral infection alone may not initiate AD in otherwise healthy patients. But, if BBB defenses fail, infection could potentially become a critical pathogenic amplifier leading to the onset of AD. However, as discussed earlier, in some cases, certain microorganisms might also enter the brain through neural pathways (with intact BBB) such as olfactory, trigeminal or even craniofacial nerves.

## 4 The third hit: neuroimmune activation and the antimicrobial amyloid response

A transformative insight in AD research was the recognition that Aβ possesses antimicrobial properties comparable to cathelicidins and defensins (Soscia et al., 2010). Aβ can bind viral and bacterial surfaces, entrap microbes via fibril formation, disrupt microbial membranes, and participate in innate immune defense pathways (Vojtechova et al., 2022).

The BBB in a healthy brain protects against pathogen entry but also limits peripheral immune cell infiltration. The brain is therefore not directly connected to the peripheral immune system but has its own specialized defenses. Microglial cells constitute the resident immune system in the CNS, detecting pathogens and other harmful molecules through pattern-recognition receptors such as TLRs and NOD-like receptors. Pathogen detection activates microglia, and persistent activation triggers the release of IL-1β and IL-18, NLRP3 inflammasome assembly, synaptic pruning, complement activation, and recruitment of peripheral immune cells through the leaky BBB. Normally, microglial activation is transient and does not cause lasting damage. However, due to chronic stimulation from prolonged BBB dysfunction and microbial exposure, microglia could shift into a damaging, pro-inflammatory phenotype that could exacerbate tau pathology, Aβ production and neuronal loss (Arber et al., 2024; Kitazawa et al., 2004; Takatori et al., 2025).

Therefore, under the proposed Triple-Hit model, Aβ deposition is not a primary pathology but initially an adaptive response to microbial presence or inflammatory stress. The third hit would, in this hypothesis, transform a short-term defensive response into long-term neurodegeneration.

## 5 Unifying the three hits into a sequential pathogenic cascade

The Triple-Hit Hypothesis proposes Alzheimer’s disease as a biologically coherent and temporally ordered cascade. In the first stage, breakdown of the BBB would expose the CNS to circulating molecules, pathogens, and immune signals normally excluded from the brain. This increased permeability would change the brain’s microenvironment and make it vulnerable to the second stage: microbial infiltration or reactivation. Viruses, bacteria, or other microorganisms persisting in peripheral tissues or within the CNS could exploit the compromised BBB (or in same cases even contribute to the disruption), providing a potent trigger for neuroimmune activation.

The third stage would follow as the response of the innate immune system. Different cells in central neural system, including microglia and neurons, would initiate the antimicrobial defense programs, part of which includes production and deposition of Aβ by neurons, which has recognized antimicrobial properties, and Tau pathology. Although initially protective, this response could become detrimential for CNS cells when sustained, driving chronic inflammation, synaptic dysfunction, progressive neuronal injury and neuronal loss, leading to the clinical signs of dementia.

This sequential framework offers a unifying explanation for several long-standing, although some speculative, observations in Alzheimer’s research. It clarifies why vascular and metabolic risk factors predict cognitive decline, why APOE4 affects both BBB and viral susceptibility, why amyloid appears late but remains a key biomarker, why infection-related diseases accelerate cognitive decline, and why anti-amyloid therapies alone have produced limited clinical benefit. In this way, the Triple-Hit Hypothesis reconciles various reports across vascular biology, infectious disease, genetics, and neuroimmunology. Furthermore, it explains why comorbidities such as diabetes, cardiovascular disease and kidney failure only present in a certain percentage of patients, as none of the three hits alone would be sufficient to trigger the development of AD.

## 6 Risk factors for AD are primarily risk factors for BBB breakdown

One of the strongest pieces of circumstantial evidence for the Triple-Hit Hypothesis is the clustering of AD risk factors around BBB-impairing conditions. Diabetes mellitus, chronic kidney disease and chronic uremia, hypertension, obesity, cardiovascular disease, traumatic brain injury, chronic inflammation, sleep disorders, aging, and also chronic infection such as gum diseases, are all known to increase BBB permeability and are also risk factors for developing AD (Biessels et al., 2008; Faraco and Iadecola, 2013; Bobot and Burtey, 2024; Galea, 2021; Han et al., 2024; Knox et al., 2022). Interestingly, there were suggestions before that AD is initially a metabolic disease, sometimes called type 3 diabetes.^1^

Conditions such as diabetes mellitus, chronic kidney disease with its associated uremic milieu, and long-standing hypertension each exert sustained vascular stress that weakens endothelial function and increases BBB permeability (Biessels et al., 2008; Bobot and Burtey, 2024). Obesity and cardiovascular disease add layers of metabolic and inflammatory strain, further undermining the barrier’s ability to regulate what enters the brain (Faraco and Iadecola, 2013). Traumatic brain injury offers a more acute but equally damaging route, often producing immediate mechanical disruption followed by prolonged secondary inflammation that continues to erode BBB integrity long after the initial event.

Even seemingly unrelated conditions, such as sleep disorders, fit neatly into this pattern. Chronic sleep deprivation undermines glymphatic clearance, elevates systemic inflammation, and has been shown to transiently open the BBB—effects that accumulate over time (Han et al., 2024). Aging, the most consistent risk factor of all, represents the slow, physiological weakening of barrier function as endothelial cells lose resilience, pericytes degenerate, and vascular inflammation gradually increases (Galea, 2021; Knox et al., 2022).

Taken together, these factors form an interesting pattern: the strongest predictors of AD seem to be the same conditions that impair the BBB. In this context, BBB breakdown might not be a secondary correlate or an incidental finding—it could be a unifying mechanism that links metabolic dysfunction, vascular compromise, inflammation, and aging into a single biological pathway leading toward brain vulnerability. This convergence might suggest a strong conceptual foundation for the idea that BBB impairment is an early and perhaps decisive event, setting the stage for subsequent microbial infiltration, neuroinflammation, and amyloid- and tau-driven pathology.

## 7 Testable predictions of the triple-hit hypothesis

The Triple-Hit Hypothesis generates several plausible predictions that are all supported by previously published research, although not all are generally accepted, especially the role of infection in the development and progression of AD. Therefore, this novel and unifying triple hit hypothesis remains speculative and will need experimental confirmation in future studies. These should include:

First, BBB dysfunction should precede both microbial/immunological insult and amyloid accumulation. Longitudinal studies should demonstrate that markers of vascular leakage—such as dynamic contrast-enhanced MRI metrics, CSF/serum albumin ratio, or circulating biomarkers of endothelial dysfunction—appear years before amyloid PET abnormalities or CSF Aβ42 decline (Nation et al., 2019; Montagne et al., 2020).

Second, microbial markers in CSF or brain tissue should be detectable primarily in individuals who already exhibit BBB leakage. This would support the concept that microbial invasion or reactivation requires a compromised barrier to access the CNS (Itzhaki et al., 1997; Readhead et al., 2018). For example, a study looking for potential brain infection in individuals with vascular diabetes could show whether brain infection occurs primarily in patients with dementia or also in patients without dementia suffering from vascular diabetes. However, it should be taken into account that some microorganisms could also enter the brain directly through nerve infections and therefore, this should be considered in such studies.

Third, antiviral or antimicrobial therapies are expected to benefit only patients with early BBB disruption as at later stages, the neural tissue would likely be so damaged it would be difficult to expect improvement with antimicrobial therapies. Stratification of clinical trials by BBB integrity could clarify inconsistencies in previous infection-related interventions for AD (Linard et al., 2020; Tzeng et al., 2018).

Fourth, therapies aimed at restoring BBB function—such as endothelial modulators, pericyte-supporting agents, or treatments targeting metabolic and vascular risk factors—should reduce downstream inflammation and amyloidogenic signaling, ultimately improving cognitive outcomes (Rust et al., 2025). Nevertheless, it should be taken into account that leaked BBB might also help in infection by allowing entrance of cytokines and perhaps antibodies into the CNS to fight infections. Therefore, such experimental treatments should be followed very closely and examine also potential detrimental effect of reducing immune response inside the CNS.

These predictions provide a clear roadmap for both experimental validation in animal models and clinical evaluation in human cohorts, highlighting the importance of temporally and mechanistically targeted interventions.

## 8 Therapeutic and diagnostic implications

The Triple-Hit Hypothesis leads to a possible broader therapeutic strategy that could be preventive rather than solely reactive. If BBB disruption initiates the cascade leading to AD, interventions that preserve or restore barrier integrity and/or therapies fighting infections at the early stage would become an important part of AD prevention and early treatment.

### 8.1 BBB-protective therapies

Potential strategies aiming to maintain BBB integrity could include modulators of endothelial function, pericyte-stabilizing treatments, inhibition of inflammatory endothelial activation, and metabolic control of glucose, insulin, and lipid levels. Endothelial modulators may improve nitric oxide signaling, reduce oxidative stress, and prevent tight-junction loss. Pericyte-stabilizing treatments, including agents that enhance PDGFRβ signaling, could counteract pericyte degeneration widely observed in early AD. Targeting inflammatory endothelial activation, for example, through TNF-α or IL-1β inhibition, may further reinforce barrier competence. Optimizing metabolic parameters also influences BBB permeability and may delay or prevent the first hit in the cascade (Whelan et al., 1980).

### 8.2 Anti-infective therapies

If microbial invasion or reactivation constitutes the second hit, reducing viral or bacterial load could interrupt disease progression. HSV-1, particularly prevalent in APOE4 carriers, is a promising target for suppressive antiviral therapy, such as acyclovir or valacyclovir. Broader-spectrum antiviral agents may be beneficial if multiple latent viruses contribute to neuroinflammation. Bacterial targets, such as lipopolysaccharide or oral pathogens like *Porphyromonas gingivalis*, could represent additional avenues for intervention as it has been suggested before (Zhao et al., 2024).

### 8.3 Immune-modulating strategies

Addressing the third hit—neuroimmune overactivation—would require modulation of the brain’s inflammatory response once microbial signals have crossed the BBB. Inhibitors of the NLRP3 inflammasome may blunt chronic neuroinflammation that accelerates amyloid production and neuronal injury. Complement pathway modulation can counteract maladaptive synaptic pruning and microglial overactivation, while reprogramming microglia from a pro-inflammatory to a neuroprotective phenotype may help resolve persistent immune activation and promote tissue repair.

### 8.4 Diagnostics

The Triple-Hit Hypothesis, if confirmed in experimental studies, could also present significant diagnostic implications. Future algorithms may integrate multiple biomarkers to detect early disease risk: imaging to assess BBB integrity (dynamic contrast-enhanced MRI, PET tracers), pathogen detection (ultrasensitive sequencing, serology, molecular assays), neuroimmune biomarkers (microglial activation, complement activity, inflammasome components), and traditional amyloid and tau measurements. Such an integrated diagnostic approach could enable interventions decades before cognitive decline, transforming AD from a late-detected neurodegenerative disease into a preventable, early-targetable disorder.

## Footnotes

1
https://www.alzheimers.org.uk/about-dementia/managing-the-risk-of-dementia/reduce-your-risk-of-dementia/diabetes
